# Electromagnetic Cell Current Modulation As Adjunctive Therapy in HIV: A Review

**DOI:** 10.7759/cureus.92697

**Published:** 2025-09-19

**Authors:** Hemant Rohera, Deepak Nagpal, Mrunali Jambhulkar

**Affiliations:** 1 Department of Research and Development, Rohera Healthcare and Technology, Pune, IND; 2 Department of Oral Pathology and Microbiology, Swargiya Dadasaheb Kalmegh Smruti Dental College and Hospital, Nagpur, IND

**Keywords:** adjunctive therapy, electromagnetic cell current modulation, hiv reservoirs, immune restoration, mitochondrial function

## Abstract

Despite the success of antiretroviral therapy (ART), people living with HIV continue to face challenges such as chronic immune activation, oxidative stress, mitochondrial dysfunction, and latent reservoirs that prevent complete immune restoration or cure. These persistent gaps highlight the research problem: the need for safe and effective adjunctive strategies that go beyond viral suppression. The objective of this review is to critically evaluate Electromagnetic Cell Current Modulation (ECCM) as a potential adjunctive therapy in HIV, integrating mechanistic evidence, preclinical data, and emerging clinical insights. Using a narrative synthesis approach, the review examines studies across cellular, animal, and early patient-level investigations, and compares ECCM with other adjunctive modalities. Key findings suggest that ECCM may modulate cytokine signaling, enhance mitochondrial activity, reduce inflammation, and potentially influence viral latency. Small pilot studies have reported improvements in CD4 counts, symptom burden, and quality of life. The implications are substantial: if validated, ECCM could complement ART by targeting host-level dysfunctions that drive morbidity. The conclusion emphasizes the urgent need for standardized protocols, rigorous animal studies, and controlled clinical trials to determine the safety, efficacy, and scalability of this treatment. Although still early in development, ECCM offers a scientifically plausible and clinically relevant avenue for future HIV care innovation.

## Introduction and background

HIV remains a significant global health challenge, and recent Joint United Nations Programme on HIV/AIDS (UNAIDS) statistics indicate that approximately 39 million people are currently living with the infection [1]. With the advent of antiretroviral therapy (ART) in the mid-1990s, the course of HIV changed dramatically, with morbidity, mortality, and viral transmission reduced substantially [2]. ART has turned HIV into a manageable disease by successfully inhibiting viral replication [3]. However, despite such progress, ART remains non-curative and does not normalize immunological balance [4]. Long-term viral control often coexists with immune dysfunction, mitochondrial dysfunction (i.e., damage to the cell’s “powerhouses” that reduces energy production), and systemic inflammation [5]. Thus, many patients on stable therapy continue to report fatigue, neuropathic pain, GI disorders, cognitive difficulties, and other symptoms that collectively compromise quality of life [6]. These persistent problems point to a critical treatment gap that cannot be closed by pharmacological regimens alone.

**Figure 1:**
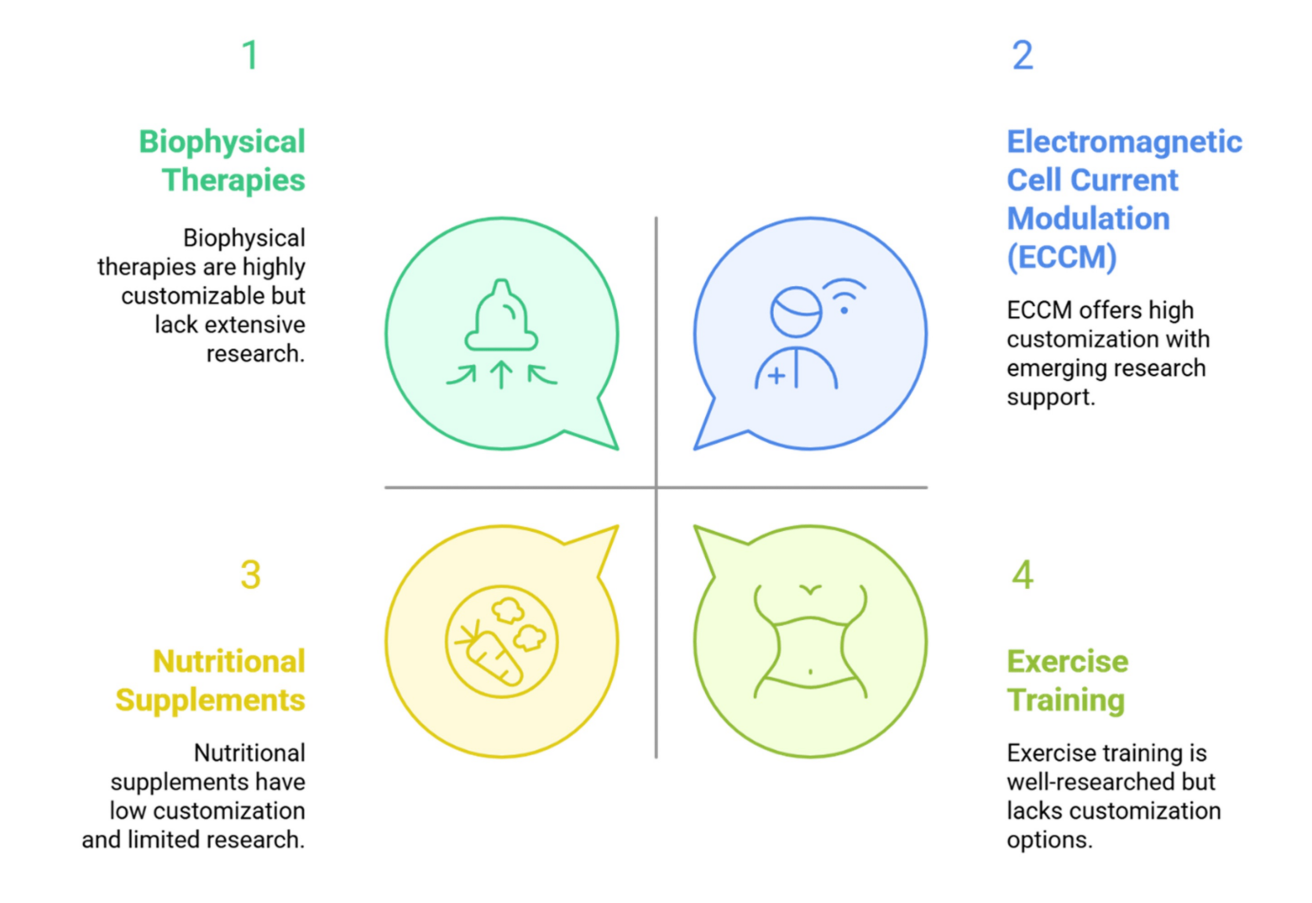
Adjunctive HIV strategies compared by customization potential and research evidence. Source: The figure was prepared by the authors using Napkin AI software.

Ongoing immune dysregulation despite successful ART has prompted investigation of add-on approaches targeting non-viral determinants of morbidity [7]. Exercise training, mindfulness-based therapies, and nutritional supplementation are among the non-pharmacological strategies explored, with varying degrees of success, for symptom management and quality of life [8]. In line with these behavioral and lifestyle interventions, biophysical therapies have also been identified as potential candidates in integrative HIV management [9]. Among these, bioelectromagnetic methods, including pulsed electromagnetic field (PEMF) therapy, have shown immunomodulatory and bioenergetic effects in various chronic and autoimmune diseases [10]. There is evidence that electromagnetic interventions can regulate cytokine activity (signaling proteins that coordinate immune responses), improve mitochondrial performance, and decrease inflammatory markers, thereby enhancing physiological function and subjective well-being [11]. As shown in Figure 1, adjunctive strategies in the management of HIV vary in their degree of customization and in the quality of supporting research.

On this basis, Electromagnetic Cell Current Modulation (ECCM) has been proposed as a new adjunctive modality for people living with HIV [12]. ECCM is a bioelectromagnetic technique that applies low-intensity electromagnetic fields to restore cellular electrical balance, enhance ion exchange, and support mitochondrial ATP production. Through the eMedica device, it is used to restore endogenous electrical potentials in cells, improve ion transfer, and increase ATP production by mitochondria [13]. Such mechanisms are particularly pertinent in HIV infection, where chronic oxidative stress and immune activation impair immune reconstitution and contribute to persistent symptom burden [14]. Limited pilot studies of ECCM suggest associations with changes in immune markers, decreased fatigue and pain, and improvements in health-related quality of life [1]. A distinguishing feature of the eMedica platform is that it provides frequency programs customizable to a patient’s symptom profile, which may be more precise and clinically feasible than other PEMF systems [15].

Although early results are promising, studies of ECCM in HIV remain in their initial phases [3]. The available research is small, often non-controlled, and has not established uniform treatment regimens regarding duration, intensity, or frequency [4]. Furthermore, direct mechanistic data on ECCM’s effects on immune restoration in HIV-positive populations are limited, and there are no data on the long-term durability of its effects. These limitations underscore the need to critically assess the current body of knowledge, place early findings in the broader context of adjunctive HIV therapies, and identify priorities to guide future studies.

This review aims to critically examine the emerging role of ECCM as an adjunctive therapy in HIV care. It seeks to synthesize the available evidence on its impact on immune restoration, symptom reduction, and patient-reported outcomes, while also considering mechanistic insights drawn from related bioelectromagnetic research. The review further evaluates the methodological strengths and weaknesses of existing studies, highlights limitations that restrict broader clinical adoption, and identifies avenues for future investigation. In doing so, it positions ECCM within the wider landscape of non-pharmacological and integrative strategies for HIV, acknowledging both its potential benefits and the rigorous evaluation still required before it can be incorporated into mainstream clinical practice.

## Review

Methods

The literature reviewed in this article covers 2015-2025, reflecting recent and clinically pertinent developments in HIV management and the growing interest in bioelectromagnetic therapies. Earlier studies on electromagnetic interventions were more general, whereas the past decade has yielded more specific work on the interface between HIV, immune restoration, and non-pharmacological adjuncts. PubMed, Scopus, and Web of Science were searched using combinations of terms such as HIV, electromagnetic cell current modulation, eMedica, pulsed electromagnetic field (EMF) therapy, and adjunctive interventions. Inclusion criteria were clinical trials, pilot studies, preclinical reports, and theoretical articles exploring immune modulation, symptom reduction, or quality of life in HIV populations or directly related bioelectromagnetic settings. Purely pharmacological studies were excluded to maintain a focus on integrative strategies.

Although we specifically focused on pilot studies of ECCM, we also incorporated studies with inconclusive or contradictory results to reduce confirmation bias and provide a balanced view. Results were synthesized narratively across three domains, mechanistic insights, preclinical models, and early clinical studies, with comparative appraisal of other adjunctive modalities. Formal statistical synthesis (e.g., meta-analysis) was not conducted because of the small number of studies, heterogeneous designs, variable endpoints (e.g., CD4 counts, cytokine markers, patient-reported outcomes), and overall study heterogeneity. Instead, a structured narrative synthesis was undertaken to trace emerging evidence, while recognizing that quantitative pooling may become feasible when more homogeneous and sufficiently powered datasets are available.

No formal risk-of-bias tool (e.g., Cochrane, ROBINS-I) was used given the preliminary and heterogeneous nature of the literature; however, each study was qualitatively assessed for methodological rigour, including sample size, design, and reported limitations. This organized narrative approach increases clarity, reduces bias, and conveys the scale, strengths, and weaknesses of the existing evidence base, while acknowledging the immature state of the field.

Principles of ECCM

EMFs are non-ionising electromagnetic energy that interact with biological tissues via electric and magnetic fields [16]. At the cellular level, numerous physiological processes depend on finely tuned electrochemical gradients, particularly transmembrane potentials that govern ion flux, signalling, and metabolism [17]. Exposure to EMFs can affect these gradients by modifying ion-channel kinetics, calcium influx, and lipid-bilayer polarization [18]. These alterations can influence cell excitability, gene expression, and redox balance [19].

EMFs already have precedent in medical applications. PEMF therapy is used in fracture non-union and has shown effectiveness in bone regeneration, osteoarthritis, and wound healing [20]. Its therapeutic actions are believed to involve modulation of signalling pathways (including MAPK and NF-κB), as well as induction of angiogenesis and mitochondrial activity [21]. Results, however, vary: biological effects depend on frequency, amplitude, waveform, and exposure duration [22]. For example, very low-frequency EMFs (<100 Hz) have been associated with increased macrophage activation, whereas higher-frequency exposures can induce oxidative stress [17].

**Figure 2:**
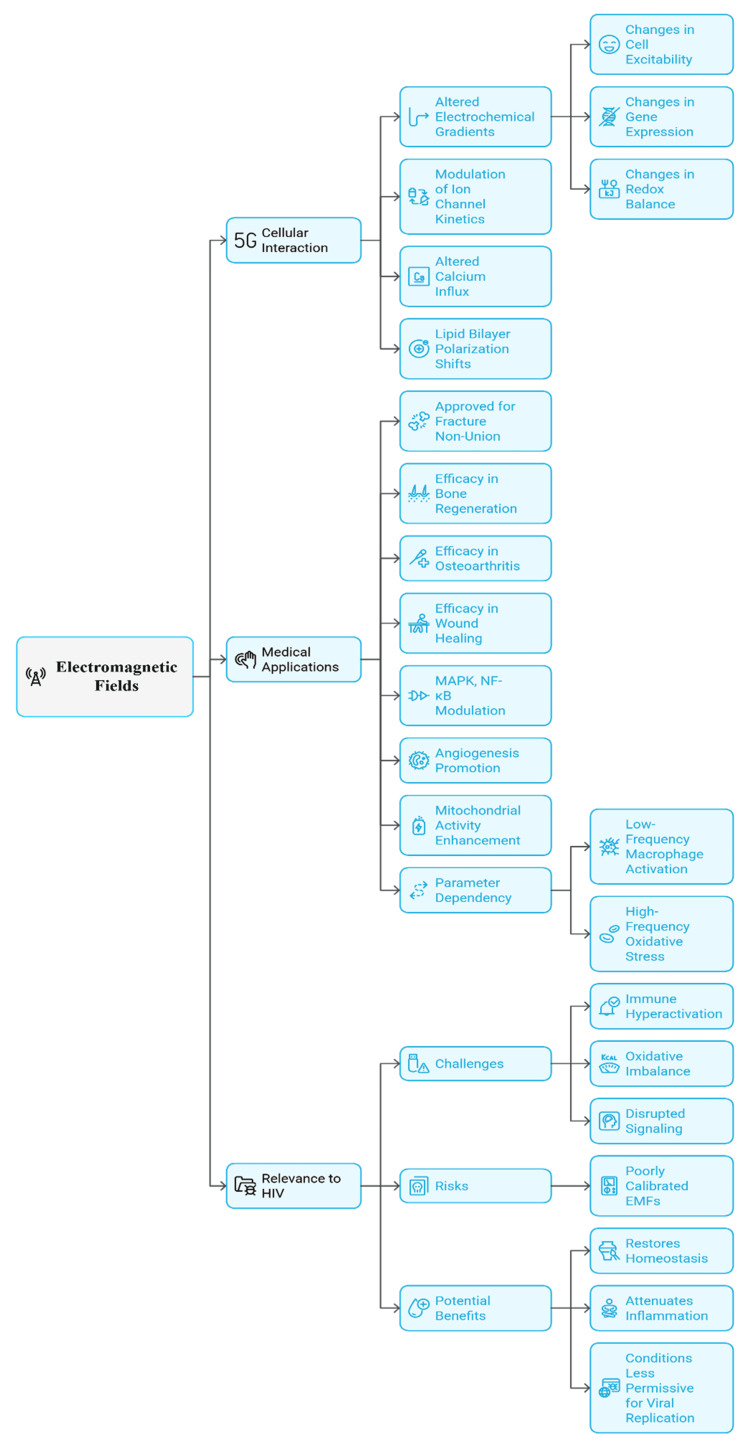
Mechanistic pathways and clinical relevance of electromagnetic fields (EMFs) in biological systems and HIV. Source: The figure was prepared by the authors using Napkin AI software.

This parameter sensitivity is particularly relevant in HIV. The infection is characterized by immune hyperactivation, oxidative stress, and dysregulated signalling; poorly tuned exposure could exacerbate pathology [23]. Conversely, carefully calibrated EMFs may promote homeostasis, reduce inflammation, and create a less permissive environment for viral proliferation [20]. The therapeutic potential of EMF therapy thus lies in targeted modulation, not indiscriminate exposure. Figure 2 illustrates EMF effects on cellular functions and clinical outcomes, which may be detrimental or therapeutic depending on the parameters used.

HIV pathophysiology and immune dysfunction

HIV pathogenesis is driven by both direct viral cytopathic effects and chronic immune dysregulation [24]. Viral entry begins when gp120 binds to CD4 receptors and chemokine co-receptors, followed by reverse transcription and integration into host DNA [25]. This process establishes both productive infection and long-lived reservoirs, particularly in memory CD4+ T cells and tissue macrophages [3]. Even with effective antiretroviral therapy (ART), these reservoirs persist and prevent eradication.

A hallmark of HIV infection is chronic immune activation. Microbial translocation from a compromised gut mucosa, ongoing antigenic stimulation, and cytokine dysregulation all contribute to systemic inflammation [26]. Persistent activation accelerates T-cell turnover, exhausts effector cells, and drives comorbidities such as cardiovascular disease and neurocognitive decline. CD4+ T-cell depletion results not only from direct viral killing but also from apoptosis and pyroptosis triggered by inflammatory mediators [27].

Although ART effectively suppresses viral replication, it does not normalize immune activation or restore full immune competence. This gap underscores the need for adjunctive interventions. EMFs, by modulating cytokine production (e.g., TNF-α, IL-10), influencing oxidative stress pathways, and enhancing mitochondrial resilience, may help attenuate the inflammatory milieu associated with HIV [28]. By recalibrating immune responses, EMF exposure could support CD4+ T-cell recovery, reduce bystander apoptosis, and potentially affect reservoir dynamics. The overlap between HIV pathophysiology and EMF effects is therefore clear: both converge on cell signaling, redox balance, and mitochondrial function. This convergence provides the rationale for exploring EMF therapy as a targeted adjunct rather than a stand-alone intervention.

Mechanistic rationale for EMF therapy in HIV

The rationale for EMF therapy in HIV rests on its ability to influence host cellular processes that intersect with viral persistence and immune dysfunction [29]. One of the most compelling aspects is its impact on intracellular signaling. EMFs can alter calcium channel activity, which modulates cascades such as NF-κB and MAPK. NF-κB is especially relevant in HIV biology because it both drives proviral transcription and sustains immune activation [30]. By fine-tuning NF-κB activity, EMF exposure could theoretically reduce the chronic inflammation caused by excessive immune activation while simultaneously limiting viral gene expression and replication [31].

Mitochondrial function is another key area of relevance. HIV infection is closely linked with mitochondrial dysfunction, which contributes to oxidative stress, energy imbalance, and premature apoptosis of immune cells [32]. Under specific conditions, EMFs have been reported to enhance mitochondrial respiration and ATP synthesis, thereby restoring energy metabolism and supporting cell survival [33]. In HIV, this could help preserve CD4+ T cells and reduce metabolic vulnerabilities that the virus exploits. Closely tied to this is the issue of redox balance [11]. Oxidative stress not only accelerates immune exhaustion but also creates a biochemical environment that favors viral transcription [7]. EMF exposure has been shown to upregulate antioxidant defenses such as superoxide dismutase and catalase, potentially reducing viral replication while maintaining immune cell function [24]. A more speculative but intriguing possibility is the effect of EMFs on HIV latency and reservoirs. Latent reservoirs remain the greatest barrier to a cure, and current strategies alternate between “shock and kill,” which reactivates latent cells for clearance, and “block and lock,” which enforces deep latency [34]. EMFs might contribute to either paradigm by modulating transcriptional pathways in latently infected cells. Whether this modulation can be controlled predictably and safely remains an open question [18].

Finally, EMF therapy must be considered alongside ART. While ART effectively suppresses viral replication, it does not address persistent metabolic dysfunction, mitochondrial injury, or immune hyperactivation [16]. EMFs could act synergistically with ART by reducing residual inflammation, supporting CD4+ T-cell viability, and potentially sensitizing reservoirs to pharmacological clearance. Evidence for such synergy exists in other chronic inflammatory and viral conditions [5], though HIV-specific validation is still needed. Taken together, these pathways present a coherent biological rationale for EMF therapy as an adjunct to ART. However, most supporting data come from indirect or non-HIV studies. Determining whether EMF-induced modulation of signaling, mitochondrial dynamics, redox balance, and latency can provide measurable clinical benefit in HIV will require rigorous preclinical validation and carefully designed clinical trials. Table 1 summarizes the potential mechanistic pathways of EMF therapy in HIV.

**Table 1:** Mechanistic pathways of electromagnetic field (EMF) therapy in HIV and their potential clinical relevance. ART: Antiretroviral Therapy; NF-κB: Nuclear Factor kappa-light-chain-enhancer of activated B cells; MAPK: Mitogen-Activated Protein Kinase; ATP: Adenosine Triphosphate; SOD: Superoxide Dismutase; CD4+ T cell: Cluster of Differentiation 4–positive T lymphocyte (helper T cell).

Mechanistic Pathway	Role in HIV Pathogenesis	Proposed Effect of EMFs	References
Intracellular signaling (NF-κB, MAPK)	NF-κB drives proviral transcription and sustains chronic immune activation.	EMFs modulate calcium channels and fine-tune NF-κB/MAPK activity → may reduce inflammation and limit viral replication.	[29]
Mitochondrial function	HIV and some ART drugs cause mitochondrial dysfunction, oxidative stress, and immune-cell apoptosis.	EMFs enhance mitochondrial respiration and ATP synthesis → support CD4+ T-cell survival and restore energy balance.	[3]
Redox balance	Oxidative stress accelerates immune exhaustion and promotes viral transcription.	EMFs upregulate antioxidant defenses (e.g., SOD, catalase) → reduce oxidative stress and preserve immune function.	[7]
Latency and reservoirs	Persistence of latent HIV reservoirs prevents a cure.	EMFs may influence transcriptional activity of latently infected cells, with potential for “shock and kill” or “block and lock” strategies.	[18]
Synergy with ART	ART suppresses viral replication but does not address immune hyperactivation or metabolic injury.	EMFs could complement ART by reducing residual inflammation, preserving CD4+ T cells, and sensitizing reservoirs to clearance.	[16]

Evidence from in vitro studies

Laboratory investigations into the effects of EMFs on biological systems provide the foundation for hypothesizing their relevance in HIV [3]. Although few experiments have directly exposed HIV-infected cells to EMFs, a substantial body of work has examined immune and epithelial cell cultures under controlled electromagnetic conditions [35]. Collectively, these studies suggest that EMFs can modulate cytokine production, oxidative-stress responses, and apoptotic pathways, all of which are central to HIV pathophysiology. Several experiments have reported anti-inflammatory effects. For example, low-frequency pulsed EMFs have been shown to reduce expression of pro-inflammatory cytokines such as TNF-α and IL-6 while enhancing anti-inflammatory mediators like IL-10 in macrophage and monocyte-derived cultures [36]. This is particularly relevant given that HIV pathogenesis is driven not only by viral replication but also by chronic immune activation. Similarly, studies on T-lymphocyte lines have demonstrated EMF-induced stabilization of mitochondrial membrane potential and decreased caspase activation, suggesting a reduction in apoptosis [37]. Because bystander T-cell apoptosis contributes significantly to CD4+ depletion in HIV, such findings could carry translational significance.

However, the literature also reports contradictory outcomes. Some studies indicate that EMF exposure can generate reactive oxygen species (ROS) rather than neutralize them, particularly at higher intensities or with prolonged durations [15]. Since oxidative stress accelerates HIV replication by activating transcription factors like NF-κB, these pro-oxidant effects could theoretically worsen disease progression. Other studies have shown no measurable immunological changes under certain exposure parameters, underscoring inconsistency across laboratories [20]. A persistent limitation of the in vitro evidence is its heterogeneity: experimental protocols vary widely in frequency (from extremely low to radiofrequency ranges), waveform (pulsed vs continuous), and exposure duration, making cross-comparison difficult. Moreover, isolated cell-culture systems cannot capture the complexity of the in vivo immune environment, including interactions among T cells, macrophages, stromal elements, and the microbiome [12]. Despite these constraints, the collective in vitro evidence suggests that EMFs can exert biologically significant, though highly context-dependent, effects on immune cells. This variability highlights both the promise and the challenge of translating EMF modulation into HIV therapy: therapeutic benefit will depend on carefully optimized parameters rather than indiscriminate application.

Evidence from animal models

Animal studies extend these observations to more complex biological systems; however, data specific to HIV are still lacking. No published studies to date have directly evaluated EMF exposure in simian immunodeficiency virus (SIV) or humanized mouse models of HIV [38]. Instead, evidence comes from research on EMFs in other viral and inflammatory contexts, as well as in tissue repair and neurodegeneration. Rodent experiments provide suggestive insights: in models of chronic inflammation, EMF exposure has been linked to reductions in systemic inflammatory cytokines, improved antioxidant enzyme activity, and enhanced macrophage function [39]. In influenza-infected mice, some studies have reported reductions in viral titers and improved survival following EMF treatment, though results have not always been reproducible [19]. These outcomes, while preliminary, align with the hypothesis that EMFs may create cellular conditions less permissive to viral propagation or more conducive to immune resilience.

The safety profile in animals has generally been reassuring, with most studies reporting no overt toxicity at therapeutic intensities [40]. Nevertheless, concerns remain about possible long-term risks, including genomic instability, reproductive effects, and exacerbation of oxidative stress at high exposure levels [41]. Given that HIV patients would likely require prolonged adjunctive therapy, long-term safety data are particularly important. A major challenge in extrapolating from these findings is the absence of HIV-specific models. Unlike acute viral infections such as influenza, HIV is characterized by chronic persistence, reservoir formation, and immune exhaustion [42]. Without targeted studies in SIV or humanized HIV models, it is impossible to determine whether the immunomodulatory and antioxidant effects of EMFs observed in rodents will translate into clinically meaningful outcomes for people living with HIV [43]. Furthermore, variability in exposure parameters across studies complicates interpretation. Until standardized protocols are tested in appropriate animal models, conclusions must remain tentative.

Clinical studies and trials

The scientific evidence base for EMF therapy in HIV is limited and methodologically weak. Some pilot studies and case series, often described as bioresonance or electromagnetic therapy, have been conducted, but none meet the standards of randomized controlled trials [44]. Other reports describe changes in surrogate markers, including increases in CD4+ T-cell counts and decreases in circulating inflammatory cytokines [45]. However, these studies were extremely small (often single- to low-double-digit sample sizes), varied substantially in endpoints and regimens, lacked appropriate controls, and inconsistently reported exposure parameters, making results difficult to interpret or compare across studies.

More substantial clinical evidence comes from related fields rather than HIV itself. EMF therapy has been explored in chronic pain syndromes, osteoarthritis, and wound healing, where it has generally been well tolerated and, in some cases, associated with anti-inflammatory or regenerative effects [18]. These findings support the safety and feasibility of EMF interventions but do not provide HIV-specific efficacy data. Notably, no clinical trials have shown that EMFs lower HIV viral load or directly affect reservoir dynamics independent of ART [46]. This suggests EMFs are unlikely to function as standalone therapies but may have potential as adjuncts to enhance immune recovery or mitigate inflammatory comorbidities.

From a comparative standpoint, other adjunctive therapies in HIV, such as nutritional supplementation, herbal medicine, and photobiomodulation, share a similar rationale of modulating host physiology rather than directly inhibiting viral replication [21]. EMFs may offer distinctive advantages in being noninvasive, reversible, and precisely calibratable by exposure parameters [7]. If future studies demonstrate reproducible benefits, e.g., reduced chronic inflammation, improved CD4+ T-cell recovery, or synergy with ART in attenuating immune activation, EMFs could occupy a meaningful niche as adjunctive therapy.

Regulatory and ethical hurdles also contribute to the paucity of high-quality trials. Non-pharmacological approaches in HIV are often met with skepticism due to historical controversies around alternative therapies. Rigorous randomized trials are needed not only to establish scientific validity but also to build credibility within the clinical community [34]. Early-phase pilot studies should prioritize well-specified endpoints (e.g., changes in immune-activation markers, oxidative-stress profiles, and patient-reported outcomes) before progressing to larger efficacy trials. In the absence of such evidence, EMF therapy will remain at the periphery of HIV research rather than entering mainstream therapeutic consideration. As summarized in Table 2, the current evidence supporting EMF therapy in HIV is limited, indirect, and methodologically weak, underscoring the need for rigorous clinical trials.

**Table 2:** Clinical evidence and limitations of EMF therapy in HIV

Domain	Findings	Limitations	References
Direct clinical studies in HIV	Small exploratory trials and anecdotal case series report modest improvements (e.g., CD4+ T-cell counts, reduced inflammatory cytokines)	Very small sample sizes, lack of controls, inconsistent exposure parameters, and poor methodological rigor	[10]
Evidence from adjacent fields	In chronic pain, osteoarthritis, and wound healing, EMF therapy is generally well tolerated and sometimes shows anti-inflammatory or regenerative effects.	Findings cannot be directly extrapolated to HIV; no evidence for effects on viral load or reservoirs.	[13]
Comparative perspective with other adjunctive therapies	EMFs share a rationale with nutritional, herbal, and photobiomodulation therapies but stand out for non-invasiveness, reversibility, and precise calibration.	Still no reproducible HIV-specific efficacy; largely theoretical without rigorous trials	[21]
Regulatory and ethical considerations	Non-pharmacological HIV interventions require high scrutiny due to historical controversies; credibility depends on well-designed trials.	Lack of randomized controlled trials; limited acceptance in mainstream HIV care	[36]
Future research priorities	Pilot studies should focus on immune activation, oxidative stress, and patient-reported outcomes before moving to large trials.	Without rigorous evidence, EMFs will remain peripheral in HIV therapy	[21]

Current evidence on EMF therapy in HIV is promising but fragmented. In vitro studies suggest potential benefits on cytokine regulation, apoptosis, and redox balance, while animal models demonstrate systemic immunomodulation and a favorable safety profile, though without addressing HIV-specific mechanisms. Clinical findings remain anecdotal, offering reassurance on safety but little efficacy data. The translational chain is therefore incomplete, with major gaps in standardized protocols, relevant animal models, and randomized trials. EMFs should be regarded as an intriguing adjunctive option that requires a systematic research program progressing from optimized in vitro HIV models to animal studies, and ultimately to rigorously designed clinical trials, to determine their true therapeutic potential.

Impact on HIV reservoirs and latency

The persistence of latent viral reservoirs is the central obstacle to eradicating HIV infection. Even under lifelong antiretroviral therapy (ART), these reservoirs, established primarily in memory CD4+ T cells and tissue macrophages, remain transcriptionally silent yet replication-competent [47]. Once therapy is interrupted, viral rebound occurs rapidly, underscoring the durability of latency. Current cure strategies largely follow two paradigms: the “shock and kill” approach, which reactivates proviruses for immune clearance, and the “block and lock” strategy, which enforces deep and durable latency. Both approaches face challenges of incomplete efficacy and toxicity [48]. EMFs present a potential, though speculative, modality for influencing reservoir dynamics. Laboratory studies in non-HIV systems show that EMF exposure can modulate transcriptional pathways such as NF-κB, CREB, and MAPK, all of which play roles in proviral gene regulation [49]. By tuning these pathways, EMFs might theoretically promote controlled reactivation of latent proviruses or suppress transcription to deepen latency. The duality of these potential effects mirrors existing cure paradigms.

At present, however, there is no direct evidence demonstrating that EMFs can reliably activate or silence latent HIV [8]. The major risk lies in unpredictable outcomes: partial reactivation could expand viral diversity or enable immune escape if clearance mechanisms are insufficient. Conversely, indiscriminate suppression might entrench reservoirs without advancing toward a functional cure. To move beyond speculation, studies must first evaluate EMFs in established latency models to determine whether consistent and controllable effects are achievable. Only through such targeted experimentation can EMFs be credibly positioned as a tool for reservoir modulation.

Synergistic potential with ART

Although ART remains the cornerstone of HIV management, it does not fully normalize immune function or eliminate reservoirs, and long-term use carries risks of metabolic toxicity, drug resistance, and adherence fatigue [49]. Adjunctive therapies are therefore evaluated not as replacements but as complements to ART. EMFs, by modulating inflammation, oxidative stress, and mitochondrial function, may offer benefits in this regard [50]. One potential synergy lies in addressing residual immune activation [7]. Even in patients with complete viral suppression, heightened inflammation contributes to comorbidities such as cardiovascular disease and cognitive decline [12]. EMF exposure has been associated with reduced pro-inflammatory cytokine production and improved antioxidant defenses in other disease contexts [18]. If translated to HIV, this effect could support more complete immune recovery and improve long-term outcomes [24].

Another area of interest is the interaction with cellular metabolism [9]. Several ART classes, particularly older nucleoside analogs, are associated with mitochondrial toxicity and oxidative imbalance [51]. Under specific parameters, EMFs have been shown to enhance mitochondrial respiration and restore redox balance [21]. This raises the possibility that EMFs could counteract ART-related toxicities, potentially allowing lower doses or reducing long-term adverse effects [30]. Yet uncertainties remain [6]. EMFs can alter membrane permeability and transporter activity, which could unpredictably affect drug uptake and distribution [14]. Without rigorous pharmacological studies, unintended interference with ART cannot be excluded [26].

**Figure 3:**
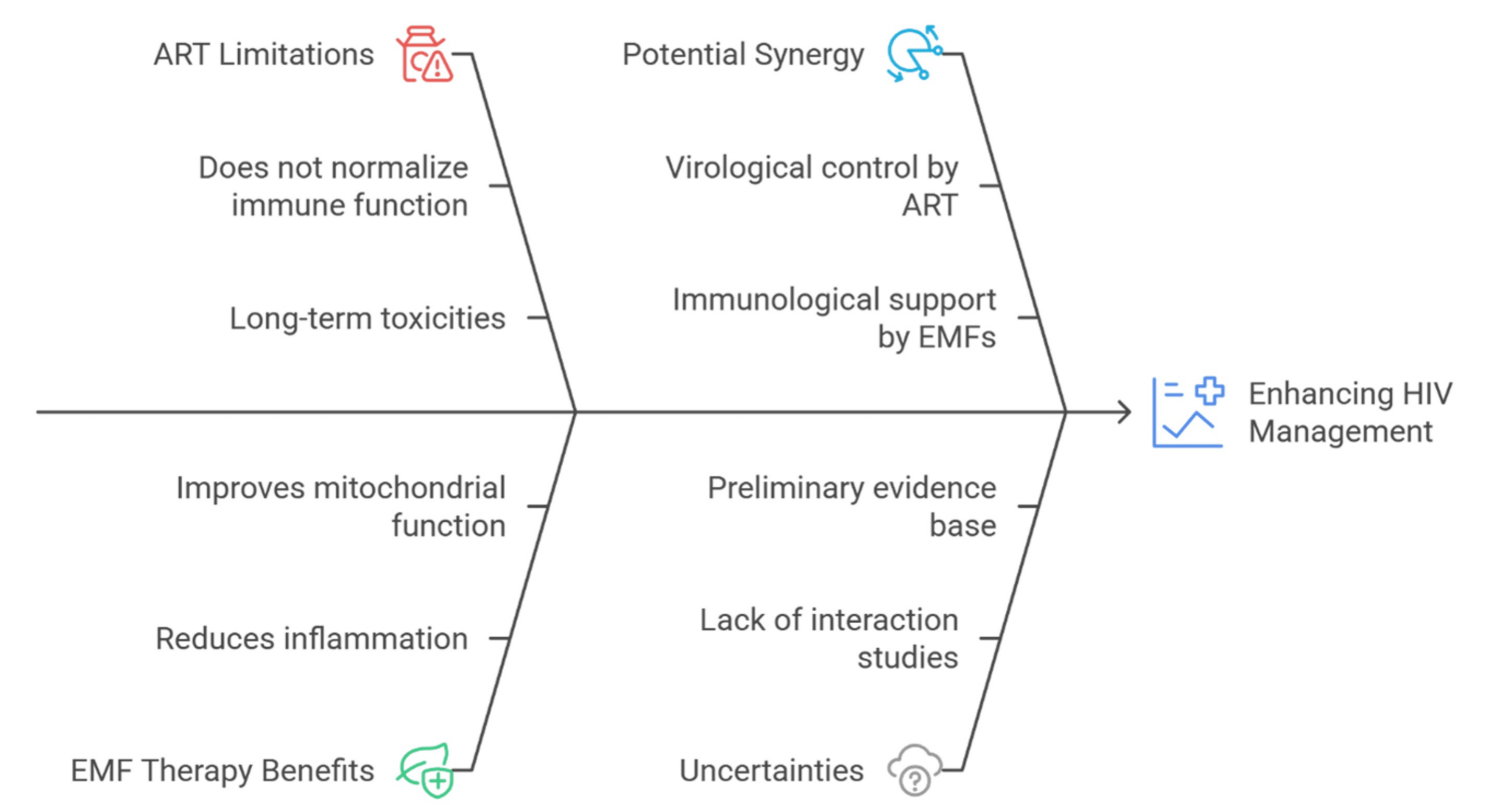
Potential synergy of ART and EMF therapy in HIV management The figure is prepared by the authors using the Napkin AI software

The potential benefits, however, are conceptually attractive: ART directly blocks viral replication, while EMFs could mitigate the immunological and metabolic disturbances that persist despite therapy [33]. Together, they might address both the virological and immunological dimensions of HIV disease [19]. Such synergy remains hypothetical but underscores the importance of structured preclinical studies to explore drug-field interactions before clinical application [27]. Figure 3 illustrates the conceptual framework in which integrating ART with EMF therapy may enhance HIV management by combining virological control with immunometabolic support, despite current uncertainties.

Safety and ethical considerations

The long-term safety of EMFs is a critical consideration for their use in HIV care [5]. Clinical applications of low-intensity EMFs in orthopedics and wound healing suggest a generally favorable safety profile, with minimal adverse events reported at therapeutic exposure ranges [11]. Nonetheless, these findings cannot be directly extrapolated to systemic and long-term use in HIV, where chronic exposure would likely be required [20]. Concerns exist in several domains [4]. First, there is the possibility of genomic instability [28]. While EMFs are non-ionizing and incapable of directly breaking DNA, some studies suggest that prolonged exposure under certain parameters could induce subtle chromosomal changes or influence DNA repair mechanisms [36]. Second, oxidative stress is a concern [13]: depending on frequency and intensity, EMFs may either enhance antioxidant defenses or promote ROS [25]. In the context of HIV, where oxidative stress is already elevated, any pro-oxidant effect could accelerate disease progression or comorbidities [31]. Finally, practical safety issues must be considered, such as interference with implanted devices like pacemakers, which is increasingly relevant in aging populations living with HIV [39].

Beyond biophysical risks, ethical considerations are paramount [8]. Electromagnetic radiation carries an undeserved reputation among the public, influenced by debates over mobile-phone safety [29]. Implementing EMFs as a medical intervention for HIV, a condition historically stigmatized and vulnerable to exploitation, requires sensitivity [35]. Clear communication of risks and benefits, strong ethical governance, and complete transparency in clinical-trial design are essential [22]. Most importantly, EMFs must not be promoted as an alternative to ART, as this could compromise adherence to life-saving therapy [32]. Their exploration should be explicitly framed as adjunctive and contingent on rigorous scientific validation [40].

Comparison with other adjunctive therapies

The limitations of ART have long motivated adjunctive strategies in HIV that aim to modulate host physiology [2]. Anti-inflammatory agents, herbal compounds, and nutritional supplementation have been tested with mixed or limited success [17]. More recently, low-level laser therapy and photobiomodulation have gained attention for effects on oxidative stress, tissue repair, and immune activation [23]. EMFs offer unique advantages, and notable challenges, within this landscape [10]. They are noninvasive, reversible, and precisely calibratable in terms of exposure parameters, which is attractive from a biomedical-engineering standpoint [16]. Unlike nutraceuticals, their action is independent of absorption or metabolic variability; unlike some herbal compounds, they are not inherently associated with drug-drug interactions with ART [34]. Compared with photobiomodulation, EMFs may penetrate deeper tissues and need not involve direct light exposure, potentially enabling systemic rather than localized effects [38].

Yet their limitations are equally clear [3]. Compared with other adjunctive strategies, evidence for EMFs in HIV is far more limited, with no large-scale trials and minimal disease-specific data [37]. Public skepticism may also be greater, influenced by cultural beliefs that electromagnetic radiation is harmful to health [1]. Finally, the requirement for specialized equipment and regulated protocols can restrict availability in resource-limited settings, where the burden of HIV is highest [30]. From a comparative perspective, EMFs should not be viewed as alternatives to proven adjunctive modalities, but rather as one option within a broader research portfolio [19]. Their specific attributes, adaptability, reversibility, and potential systemic effects, warrant further exploration, but only alongside rigorous assessment of cost-effectiveness, scalability, and context-specific suitability [28]. Table 3 indicates that adjunctive strategies in HIV offer varied benefits, with EMFs showing unique potential but limited disease-specific evidence.

**Table 3:** Comparative evaluation of adjunctive strategies in HIV management. ART: Antiretroviral Therapy; EMFs: Electromagnetic Fields; EM (radiation): Electromagnetic (radiation).

Adjunctive Strategy	Strengths / Advantages	Limitations / Challenges	References
Nutritional supplementation	May improve general health and immune support.	Benefits inconsistent; low customization; absorption/metabolic variability.	[17]
Herbal compounds	Potential anti-inflammatory or immunomodulatory effects.	Risk of drug-drug interactions with ART; variable potency and quality.	[2]
Anti-inflammatory agents	Can target residual inflammation and immune activation.	Modest benefits; risk of side effects with long-term use.	[25]
Low-level laser therapy / Photobiomodulation	May reduce oxidative stress, aid tissue repair, and modulate immune function.	Limited tissue penetration; localized rather than systemic effects.	[38]
Electromagnetic fields (EMFs)	Noninvasive, reversible; precise parameter calibration; deeper tissue penetration; potential systemic effects.	Weak HIV-specific evidence; no large-scale trials; skepticism related to EM-radiation concerns; specialized equipment limits accessibility.	[30]

Limitations and future directions

The literature on EMFs as an adjunctive therapy in HIV is dispersed. Most work has been conducted in in vitro systems or non-HIV models, with virtually no studies in simian or humanized models, both critical for understanding latency and reservoirs. Clinical studies are small and poorly controlled, offering largely anecdotal information. A major limitation is the absence of standardized procedures: exposure frequencies, intensities, and durations vary widely, making replication and synthesis difficult. Additional uncertainty stems from methodological issues such as the adequacy of sham controls. Although EMFs appear safe in orthopedic and wound-healing indications, the effects of long-term, systemic application in immunocompromised individuals have not been adequately addressed. Ethical concerns also exist regarding the potential misrepresentation of EMFs as an alternative to proven antiretroviral therapy.

A further limitation of this review is the absence of formal statistical synthesis (e.g., meta-analysis). The available literature is insufficient, heterogeneous, and imprecise in endpoints (e.g., CD4 counts, cytokine markers, patient-reported outcomes), precluding meaningful pooling of effect estimates. This reflects the nascent state of the evidence base rather than methodological oversight. Quantitative techniques (e.g., meta-analysis, meta-regression) will be needed to strengthen conclusions and inform trial design as more homogeneous and adequately powered studies become available.

Standardization of ECCM protocols should be a primary focus of future research, with explicit specification of frequency, field strength, waveform, session duration, and total dose to facilitate replication and cross-study comparison. Initial efforts should validate effects in appropriate animal models before proceeding to clinical trials. Early clinical work should prioritize endpoints beyond CD4 counts, such as markers of inflammation, oxidative stress, and reservoir dynamics. Long-term safety surveillance, rigorous statistical analysis, and interdisciplinary collaboration will be essential to advance EMFs from a preliminary concept to a plausible adjunctive therapy.

## Conclusions

This review synthesizes existing evidence on ECCM as an adjunctive therapy for HIV, integrating mechanistic theory, preclinical findings, and early clinical experience into a single model. It examines how ECCM may affect immune activation, oxidative stress, mitochondrial biology, and latency, presenting a multidimensional rationale that has not otherwise been explored in an integrated fashion. Rather than treating electromagnetic therapies or other adjunctive measures in isolation, the review situates ECCM within the broader context of HIV treatment, highlighting strengths such as noninvasiveness, reversibility, and tunability, while contrasting these with the limitations of other supportive approaches. It also emphasizes key uncertainties, including the narrow scope of existing evidence and methodological heterogeneity across studies. A major contribution is the delineation of a translational pathway for ECCM research, progressing from in vitro studies and animal models to controlled clinical trials. Priority areas include standardizing exposure protocols, determining effects on viral reservoirs, evaluating potential synergy with ART, and establishing long-term safety. By also addressing practical considerations such as scalability and cost-effectiveness, the review offers a prospective agenda for investigators, clinicians, and policymakers. In doing so, it lays the foundation for future assessment of ECCM’s contribution to HIV care. Next steps include randomized controlled trials using standardized ECCM protocols, studies of pharmacologic interactions with ART, incorporation of validated patient-reported outcomes, and long-term safety monitoring to define ECCM’s role in HIV management.
